# Satiety Hormone LEAP2 After Low-Calorie Diet With/Without Endobarrier Insertion in Obesity and Type 2 Diabetes Mellitus

**DOI:** 10.1210/jendso/bvae214

**Published:** 2024-12-04

**Authors:** Mimoza Emini, Raghav Bhargava, Madhawi Aldhwayan, Navpreet Chhina, Marcela Rodriguez Flores, Ghadah Aldubaikhi, Moaz Al Lababidi, Werd Al-Najim, Alexander D Miras, Aruchuna Ruban, Michael A Glaysher, Christina G Prechtl, James P Byrne, Julian P Teare, Anthony P Goldstone

**Affiliations:** PsychoNeuroEndocrinology Research Group, Division of Psychiatry, Department of Brain Sciences, Imperial College London, Hammersmith Hospital, London W12 0NN, UK; PsychoNeuroEndocrinology Research Group, Division of Psychiatry, Department of Brain Sciences, Imperial College London, Hammersmith Hospital, London W12 0NN, UK; College of Applied Medical Sciences, King Saud University, Riyadh 11451, Saudi Arabia; PsychoNeuroEndocrinology Research Group, Division of Psychiatry, Department of Brain Sciences, Imperial College London, Hammersmith Hospital, London W12 0NN, UK; PsychoNeuroEndocrinology Research Group, Division of Psychiatry, Department of Brain Sciences, Imperial College London, Hammersmith Hospital, London W12 0NN, UK; PsychoNeuroEndocrinology Research Group, Division of Psychiatry, Department of Brain Sciences, Imperial College London, Hammersmith Hospital, London W12 0NN, UK; PsychoNeuroEndocrinology Research Group, Division of Psychiatry, Department of Brain Sciences, Imperial College London, Hammersmith Hospital, London W12 0NN, UK; Department of Metabolism, Diabetes and Reproduction, Imperial College London, Hammersmith Hospital, London W12 0NN, UK; Department of Metabolism, Diabetes and Reproduction, Imperial College London, Hammersmith Hospital, London W12 0NN, UK; Department of Surgery and Cancer, Imperial College London, St. Mary‘s Hospital, London W2 1NY, UK; Division of Surgery, University Hospital Southampton NHS Foundation Trust, Southampton SO16 6YD, UK; Clinical Trials Unit, Department of Public Health, Imperial College London, London W12 7TA, UK; Division of Surgery, University Hospital Southampton NHS Foundation Trust, Southampton SO16 6YD, UK; Department of Surgery and Cancer, Imperial College London, St. Mary‘s Hospital, London W2 1NY, UK; PsychoNeuroEndocrinology Research Group, Division of Psychiatry, Department of Brain Sciences, Imperial College London, Hammersmith Hospital, London W12 0NN, UK

**Keywords:** LEAP2, obesity, insulin, glucose, appetite, weight loss, duodenal-jejunal bypass liner

## Abstract

**Context:**

The liver/foregut satiety hormone liver-expressed antimicrobial peptide 2 (LEAP2) is an inverse agonist at the acyl ghrelin receptor (GHSR), increasing after food intake and decreasing after bariatric surgery and short-term nonsurgical weight loss, but effects of long-term dietary weight loss are unknown.

**Objective:**

The objective of this study was to examine and compare the effects of these interventions on fasting and postprandial plasma LEAP2 and investigate potential metabolic mediators of changes in plasma LEAP2.

**Methods:**

Plasma LEAP2 was measured in a previously published 2-year trial comparing standard medical management (SMM) (including 600-kcal/day deficit) with duodenal-jejunal bypass liner (DJBL, Endobarrier) insertion (explanted after 1 year) in adults with obesity and inadequately controlled type 2 diabetes mellitus.

**Results:**

In the SMM group (n = 25-37), weight decreased by 4.3%, 8.1%, 7.8%, and 6.4% at 2, 26, 50, and 104 weeks and fasting plasma LEAP2 decreased from baseline mean ± SD 15.3 ± 0.9 ng/mL by 1.7, 3.8, 2.1, and 2.0 ng/mL, respectively. Absolute/decreases in fasting plasma LEAP2 positively correlated with absolute/decreases in body mass index, glycated hemoglobin A_1c_, fasting plasma glucose, serum insulin, homeostatic model assessment for insulin resistance, and serum triglycerides. Despite greater weight loss in the DJBL group (n = 23-30) at 26 to 50 weeks (10.4%-11.4%), the decrease in fasting plasma LEAP2 was delayed and attenuated (vs SMM), which may contribute to greater weight loss by attenuating GHSR signaling. Plasma LEAP2 did not increase with weight regain from 50 to 104 weeks after DJBL explant, suggesting a new set point with weight loss maintenance. Increases in plasma LEAP2 after a 600-kcal meal (10.8%-16.1% at 1-2 hours) were unaffected by weight loss, improved glucose metabolism, or DJBL insertion (n = 9-25), suggesting liver rather than duodenum/jejunum may be the primary source of postprandial LEAP2 secretion.

**Conclusion:**

These findings add to our understanding of the regulation and potential physiological role of plasma LEAP2.

Obesity is a chronic relapsing disease, associated with substantial societal and economic effects. It is a leading cause of premature death and serious health burden [1]. Several methods to address weight management have been tried with varied success, including caloric restriction, bariatric surgery, and newer licensed oral/injectable medications such as glucagon-like peptide-1 (GLP-1) and glucose-dependent insulinotropic polypeptide receptor analogues [2]. Identification of further therapeutic targets that improve weight loss and more importantly maintain this reduction in obesity are needed, especially as some people do not wish to have surgery, cannot tolerate these medications, have weight loss that may not normalize body composition, and increasingly combination therapies targeting gut hormones are being used to improve weight loss outcomes.

Ghrelin is a stomach-derived orexigenic hormone that stimulates appetite and food intake as its active form acyl ghrelin (AG) via the growth hormone secretagogue receptor (GHSR) in the vagus nerve, hypothalamus, brainstem, and brain reward circuits [3-9]. AG also increases plasma growth hormone, cortisol and glucose, and reduces insulin secretion [10-12].

In rodents and humans, states of negative energy balance, including fasting and weight loss from caloric dietary restriction (low-calorie (LCD) or very low-calorie diet (VLCD)), increase plasma AG, while food intake and obesity reduce plasma AG [11, 13-20]. Indeed, plasma total ghrelin remains elevated even at 1 year after cessation of an eight-week VLCD for obesity [6]. Rodent studies support a role for the increase in plasma AG induced by weight loss in weight regain after cessation of caloric restriction [21]. In humans, there is variable evidence that increases in plasma total ghrelin or AG after diet-induced weight loss predicts subsequent weight regain [22-26].

Liver-expressed antimicrobial peptide 2 (LEAP2) is a hormone with identical structure in mice and humans, expressed in the liver, jejunum, and duodenum [27-30]. Recently, in preclinical studies, LEAP2 has been identified as a competitive AG antagonist and inverse agonist at the constitutively active GHSR [17, 31-33]. Plasma LEAP2 is regulated in a reciprocal manner to AG, that is, decreases with fasting/weight loss, and increases with refeeding/weight gain in rodents and humans [17, 31-36]. In mice, peripheral and central LEAP2 administration antagonizes orexigenic effects of AG administration, and on its own reduces food intake [36-39], and a long-acting LEAP2 peptide reduces weight hepatic steatosis over four weeks in diet-induced obesity [40]. *Leap2* gene deletion enhances the orexigenic effects of exogenous AG in mice [41].

Intravenous LEAP2 administration in men without obesity reduced ad libitum food intake without side effects, though without any reduction in appetite ratings [36]. In adults without obesity, postprandial increases in LEAP2 correlated positively with postprandial decreases in appetite and food cue reactivity to high-energy foods, with a trend for decreases in ad libitum food intake [42], while in adults with overweight/obesity fasting plasma LEAP2 negatively correlated with hunger ratings [43]. These data point to plasma LEAP2 being an important satiety signal by modifying GHSR signaling in humans.

Regulation of fasting and postprandial increases in plasma LEAP2 is not fully understood, and it remains unclear from rodent, human, and organoid studies whether liver, duodenum, or jejunum are differential sources of plasma LEAP2, especially postprandially [30, 39, 44, 45]. Similarly, human studies correlating fasting and postprandial increases in plasma LEAP2 with fasting and postprandial increases in metabolic markers often suggest potential roles for blood glucose, insulin, and/or triglycerides in the stimulation of LEAP2 secretion [17, 42, 43, 46-48], while effects of exercise and ketone administration in rodents and humans have suggested that β-hydroxybutyrate may inhibit hepatic LEAP2 secretion [44, 49, 50]. Other possible mediators for postprandial increases in plasma LEAP2 could be direct contact of nutrients with intraluminal surfaces of the duodenum/jejunum or portal venous delivery of glucose, triglycerides, or other metabolites to the liver. Indeed, the greater postprandial release of plasma LEAP2 after oral ingestion than intravenous administration of glucose implicates an “incretin” effect [50].

In humans, weight loss after Roux-en-Y gastric bypass (RYGB) and vertical sleeve gastrectomy (VSG) surgery for obesity reduced fasting and/or postprandial plasma LEAP2 [17]. Short-term, 2-week LCD also reduced fasting plasma LEAP2 in obesity, though plasma LEAP2 tended to increase post glucose ingestion [51]. However, the effects of long-term dietary caloric restriction for obesity causing weight loss on plasma LEAP2, and the potential metabolic mediators, are unknown in humans.

Insertion of a duodenal-jejunal bypass liner (DJBL, Endobarrier device) is a nonsurgical endoscopic intervention in which a 60-cm plastic tube lines the proximal small bowel, mimicking the foregut effects of RYGB surgery, as food is not in contact with the foregut after ingestion. DJBL insertion has greater weight loss and often superior improvement in glycemic control (vs LCD and standard medical management [SMM] alone), in adults with obesity and inadequately controlled T2DM [52-54], and in our recent phase 2 clinical trial [55-59], and improvement in nonalcoholic steatohepatitis [60, 61]. Examining the effects of DJBL insertion on plasma LEAP2 enables comparison of the role of duodenal/jejunal vs hepatic origins of LEAP2 as food is excluded from contact with the former.

Therefore, the hypotheses for the present study were that in adults with obesity, long-term weight loss through caloric restriction will (i) decrease fasting and/or postprandial plasma LEAP2, (ii) which will positively correlate with decreases in weight, improvements in glucose and/or fat metabolism, while (iii) weight regain will increase fasting plasma LEAP2, and (iv) DJBL insertion will exaggerate the decrease in fasting and/or postprandial plasma LEAP2 due to loss of contact of ingested food with the duodenum/jejunum.

Hence, plasma LEAP2 was measured in a secondary analysis of our previously published phase 2 clinical trial comparing effects of SMM including caloric restriction and increased physical activity, without or with DJBL insertion over 12 months, with a further 12-month follow-up after DJBL removal, in adults with obesity and T2DM [55-59]. Participants had multiple visits after an overnight fast, with a subgroup also consuming a fixed meal, with measurement of fasting and postprandial plasma LEAP2 and glucose, serum insulin and triglycerides, and glycated hemoglobin A_1c_ (HbA_1c_).

## Materials and Methods

### Study Design

Participant data are taken from a randomized, controlled interventional clinical trial, in a cohort of patients with obesity (body mass index [BMI] 30-50 kg/m^2^) and inadequately controlled T2DM (HbA_1c_ 7.7%-11.1% or 58-97 mmol/mol, on oral hypoglycemic medication only) [55, 56, 59]. Participants recruited received standard medical management (SMM) for obesity and T2DM including dietary restriction, increased physical activity, and escalation of T2DM medication following American Diabetes Association guidelines [62], alone or in addition to an endoscopic insertion of a DJBL Endobarrier device, as in the original protocol [55]. This involved advice to follow a daily LCD of 1200 to 1500 kcal for women and 1500 to 1800 kcal for men, eat regularly every day (5 times/day), control portion sizes and intake of carbohydrates/starchy foods, increase intake of low glycemic index and high-protein foods, and vegetables, and reduce intake of foods high in fat, sugar, and alcohol. Participants were advised to include more physical activity in their daily routine, for example, to walk more every day, with a goal of 2.5 hours a week of moderate intensity and 75 minutes a week of vigorous intensity aerobic activity, and muscle strengthening activities more than 2 days a week.

Participants were further split into mechanistic subgroups, included in this analysis, undergoing functional magnetic resonance imaging or taste experimental visits (Supplementary Fig. S1 [63]).

Data are taken from clinical visits at −2, 2, 26, 50, and 104 weeks. Participants arrived after an overnight fast for each visit, with the implant of the DJBL device at week 0 and explant at 52 weeks. Visit at −2 weeks consisted of baseline measurements including blood work, anthropometry (BMI, body fat percentage), and record of eating habits, diet, and lifestyle (Supplementary Fig. S1 [63]). Visits at 2, 26, 50, and 104 weeks were follow-ups to the treatment intervention (see Supplementary Fig. S1 [63]).

A sweet taste detection task was performed in the taste mechanistic subgroup only at weeks −2, 2, and 26, in which 7 incremental sucrose concentrations were used to examine sweet taste detection thresholds, before the fasting blood samples were taken (group included as covariate in correction of fasting and postprandial outcomes; see Supplementary Methods 2.4 [63]) [55, 56, 59].

### Fixed Test Meal

In the subgroup examining postprandial changes, participants were given a fixed meal consisting of 250-mL vanilla Fortisip Compact milkshake (Nutricia), containing 600 kcal (carbohydrates 49%, fat 35%, protein 16%), at 1.5 hours after starting the sweet taste task, at the −2, 2, 26, and 50 week visits, with postprandial blood samples taken at *t* = 0, 60, and 120 minutes.

### Inclusion and Exclusion Criteria for Endobarrier Study

For summary of inclusion and exclusion criteria for participants, see Supplementary Methods 2.2 [63] with a complete list in the protocol papers [55, 56].

### Blood Collection, Storage, and Assay

Fasting blood samples were taken at the −2, 2, 26, 50, and 104 week visits, with postprandial samples collected at all visits except for week 104. Plasma LEAP2 concentrations were measured using a commercial enzyme immunoassay (EK-075-40, Phoenix Pharmaceuticals Inc) according to the manufacturer's instructions, which has been previously validated using human samples [17, 42]. For further details, including measurement of plasma glucose, serum insulin and triglycerides, and HbA_1c_, see Supplementary Methods 2.3 [63].

### Appetite Visual Analogue Scale Ratings

Serial ratings of hunger, fullness, pleasantness to eat, volume of food able to eat were recorded on a 100-mm visual analogue scale (VAS) using a tablet computer at each visit. Composite appetite was calculated by combining these ratings: appetite = [hunger + pleasantness + volume + (100—fullness)]/4 [42].

### Statistical Analysis

All statistical analyses were performed using IBM SPSS version 29 and GraphPad Prism v9.5.1 with effects considered statistically significant at *P* less than .05.

Results for some of the clinical and appetite outcomes, with comparison between groups, from the trial have already been published in several papers, including for BMI, weight loss, HbA_1c_, fasting plasma glucose, serum insulin and triglycerides, fasting and postprandial hunger, fullness, and composite appetite ratings [56-59]. These longitudinal results are also included in the present analysis, as the included participants differ from that previously published, since in this report outcomes were analyzed only from trial participants in the functional neuroimaging and taste mechanistic subgroups 1 and 3, and only in those who had plasma LEAP2 measurements available at baseline and at least at 1 follow-up visit (n = 67 out of n = 113 total in the primary analysis population). This allows the combination of results across mechanistic subgroups 1 and 3, and the appropriate direct visualisation, comparison, and statistical corelation of these clinical outcomes with plasma LEAP2.

#### Longitudinal analysis of study outcomes

##### Anthropological measures

Demographic data are presented as mean ± SEM or median (interquartile range, IQR) if not normally distributed (Table 1). A mixed-effect model was performed with group (SMM vs DJBL) as between-participant factor, and weeks as a within-participant factor, to examine statistically significant changes in BMI, percentage weight loss, HbA_1c_, appetite, hunger and fullness ratings, and current use of each diabetes drug class and β-blockers encoding “Yes” as 1 and “No” as 0, at each visit (−2 to 104 weeks) (Supplementary Fig. S8; Supplementary Tables S3, S4 [63]). Post hoc analyses using the Fisher least significant difference (LSD) test examined differences from baseline visit (−2 weeks) in each group and between groups at each visit.

**Table 1.** Participant characteristics at baseline visit

		Fasting cohort					Postprandial subcohort				
					DJBL vs SMM					DJBL vs SMM	
		All	SMM	DJBL	test statistic	*P*	All	SMM	DJBL	test statistic	*P*
**N**		67	30	37			40	17	23		
**Age, y**	**Mean** ± **SD** **Median (IQR)**	54 (48-58)	54 (49-59)	52 (47-58)	*U* 759	.81	53 ± 6.9	55 ± 4.8	52 ± 8.0	*t* −1.08	.29
	**range**	32-64	31-64	33-64			34-64	48-64	34-64		
**Female**	**n (%)**	33 (43.4%)	15 (44.1%)	18 (42.9%)	χ^2^ 0.06	.80	20 (43.5%)	11 (55.0%)	9 (33.3%)	χ^2^ 2.74	.098
**White ethnicity**	**n (%)**	55 (72.4%)	23 (67.6%)	32 (76.2%)	χ^2^ 1.03	.31	38 (82.6%)	16 (84.2%)	22 (81.5%)		≥.999
**Weight, kg**	**Mean** ± **SD**	106.0 ± 16.9	102.5 ± 14.0	108.9 ± 18.6	*t* 1.43	.16	105.9 ± 17.9	99.4 ± 13.9	110.5 ± 19.2	*t* 1.72	.093
	**range**	66-153	66-132	73-153			66-150	66-123	73-150		
**BMI, kg/m^2^**	**Median (IQR**)	35.4 (32.6-39.4)	34.9 (31.8-38.8)	36.3 (32.8-39.8)	*U* 661	.45	34.9 (32.2-39.0)	35.1 (31.9-38.5)	34.0 (32.3-39.3)	*U* 266	.83
	**Range**	29-51	29-44	29-51			29-48	29-44	29-48		
**T2DM**	**n (%)**	76 (100%)	34 (100%)	42 (100%)			46 (100%)	19 (100%)	27 (100%)		
**HbA_1c_, mmol/mol**	**Median (IQR)**	61 (53-73)	59 (52-67)	63 (55-75)	*U* 12214	**.005*^c^***	73 (62-82)	64 (58-87)	75 (66-82)	*U* 226	.29
**Fasting glucose, mmol/L**	**Mean** ± **SD**	10.1 ± 2.7	9.3 ± 2.4	10.7 ± 2.9	*t* 2.09	**.040*^a^***	10.9 ± 2.6	10.2 ± 2.5	11.5 ± 2.6	*t* 1.45	.15
**Fasting insulin, mU/L**	**Median (IQR)**	9.7 (6.7-15.9)	9.7 (6.7-18.1)	9.2 (6.7-14.6)	*U* 738	.39	9.7 (6.7-16.2)	9.9 (6.1-18.7)	9.7 (7.0-15.4)	*U* 232	.87
**HOMA-IR**	**Median (IQR)**	4.7 (3.2-6.5)	4.2 (2.7-7.8)	4.9 (6.1-3.6)	*U* 602	.52	4.9 (3.3-5.9)	4.6 (2.2-9.1)	4.9 (3.8-5.9)	*U* 203	.59

Participants characteristics at baseline visit (−2 weeks) both in DJBL and SMM with plasma samples available when fasting and in postprandial subcohort. Characteristics of participants with continuous variables denoted as mean ± SD or median and interquartile range (IQR) if not normally distributed with range, or n (%). Comparisons made using, independent samples *t* test, *t* test statistic; Mann-Whitney *U* test, *U* statistic, and chi-square test, χ^2^ statistic.

Ranges: HbA_1c_ normal 20 to 41 mmol/mol; fasting glucose greater than 7.0 mmol/L diagnostic of T2DM; fasting insulin normal 3 to 15 mU/L; HOMA-IR optimal less than 1, greater than 1.9 early insulin resistance, greater than 2.9 marked insulin resistance.

Abbreviations: BMI, body mass index; DJBL, duodenal-jejunal bypass liner; HbA_1c_, glycated hemoglobin A_1c_; HOMA-IR, homeostasis model assessment for insulin resistance; SMM, standard medical management; T2DM, type 2 diabetes mellitus.

*P* values in bold indicate statistical significance. *^a^P* less than .05. *^b^P* less than .01. *^c^P* less than .005. *^d^P* less than .001.

##### Fasting analysis

Longitudinal data from the Endobarrier study were analyzed using a mixed-effects model to examine changes in outcome variables. Analysis included group (SMM vs DJBL) as a between-participant factor, and weeks as a within-participant factor. Post hoc analyses using the Fisher LSD test examined differences from baseline visit (−2 weeks) in each group and between groups at each visit. This analysis was also run separately for each group to identify any within-group changes in outcomes measured.

##### Postprandial analysis

Identical analyses were performed in the subgroup having a fixed meal using the incremental area under the curve between 0 and 120 minutes (iAUC_0-120min_). Comparisons between time points (*t* = 0, 60, and 120 minutes) for increase in postprandial plasma LEAP2 was made using a general linear model including time as a within-participant factor, with a post hoc Fisher LSD test at baseline visit (−2 weeks) only.

For further details, see Supplementary Methods 2.6.1 [63].

#### Variables correlating with absolute and changes in plasma LEAP2

##### Fasting analysis

A linear mixed-effect model analysis was performed to examine the relationships between absolute and changes from baseline visit (−2 weeks) in outcome variables (BMI, percentage weight, glucose, HbA_1c_, insulin, HOMA-IR, triglycerides, appetite, and hunger ratings) with absolute and changes from baseline visit (−2 weeks) of fasting plasma LEAP2. Analysis was conducted separately for the DJBL group from −2 to 50 weeks and the SMM group from −2 to 104 weeks, reporting the pseudo-*r*^2^ value, slope (β), and *P* value. This was also performed both with the DJBL and SMM groups combined to look for significant group × independent variable interactions indicating differences in the slopes between groups.

##### Duodenal-jejunal bypass liner explant subgroup

To examine the effect of weight regain and changes in metabolism with changes in plasma LEAP2 after DJBL explant, a correlation analysis was performed to examine the relationships between outcome variables (weight, fasting glucose, HbA_1c_, insulin, HOMA-IR, triglycerides, hunger, and appetite) and changes in fasting plasma LEAP2, in the DJBL group from 50 to 104 weeks, reporting Pearson (*r*_P_), or Spearman (*r*_S_) if not normally distributed, correlation coefficients

##### Postprandial analysis

Identical analyses to the fasting analyses were performed in the sub-group having the fixed meal to examine the relationships between absolute and changes from baseline visit (−2 weeks) in outcome variables (BMI, percentage weight, HbA_1c_, postprandial iAUC_0–120min_ glucose, appetite, and fullness ratings) with absolute and changes from baseline visit (−2 weeks) of postprandial iAUC_0–120min_ plasma LEAP2.

For further details, see Supplementary Methods 2.6.2 [63].

## Results

### Demographics of Participants

Participant demographics are presented in Table 1. Analysis in the entire Endobarrier study cohort used fasted outcomes, while analysis on the taste subgroup cohort used postprandial outcomes. Reported outcomes differ from the previously published Endobarrier papers [56-59], as participants are only included who have plasma LEAP2 measurements available.

For the Endobarrier study, at baseline the SMM and DJBL groups had similar sex distribution, ethnicity, BMI, HbA_1c_, fasting insulin, and HOMA-IR; however, fasting glucose was higher in the DJBL than in the SMM group in the whole Endobarrier study (but not taste subgroup) (Supplementary Tables S3, S4 [63]).

There was no visit × group interaction (*P* = .34-.64), nor main effect of group (*P* = .14-.96), on use of any glucose-lowering medication group or β-blockers, though there was the expected greater use of proton pump inhibitors (PPIs) from 2 to 50 weeks in the DJBL group (Supplementary Fig. S8, Supplementary Tables S3, S4 [63]), as per study protocol [55, 56, 59].

Within- and between-group effects of the SMM and DJBL interventions on BMI, body weight, HbA_1c_, fasting plasma glucose, HOMA-IR, serum triglycerides, and hunger, fullness, and appetite VAS ratings are essentially identical in direction for the subgroup in the present analysis with plasma LEAP2 available, as for the complete primary analysis population and individual mechanistic subgroup analyses [56-59], and are included to enable direct comparison with plasma LEAP2.

### Longitudinal Outcomes

#### Longitudinal changes in body mass index and weight

##### Standard medical management group

**Figure 1.**
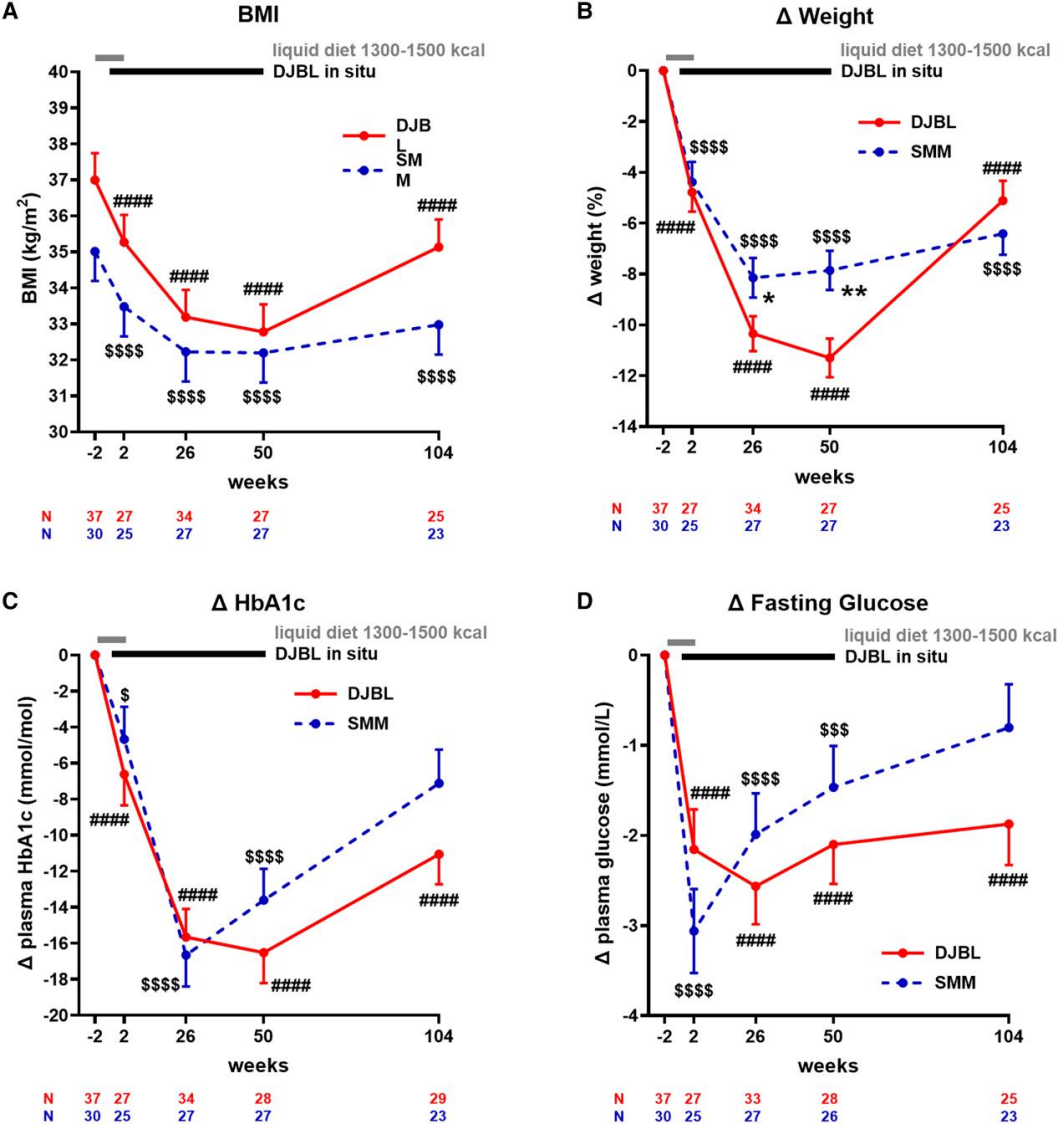

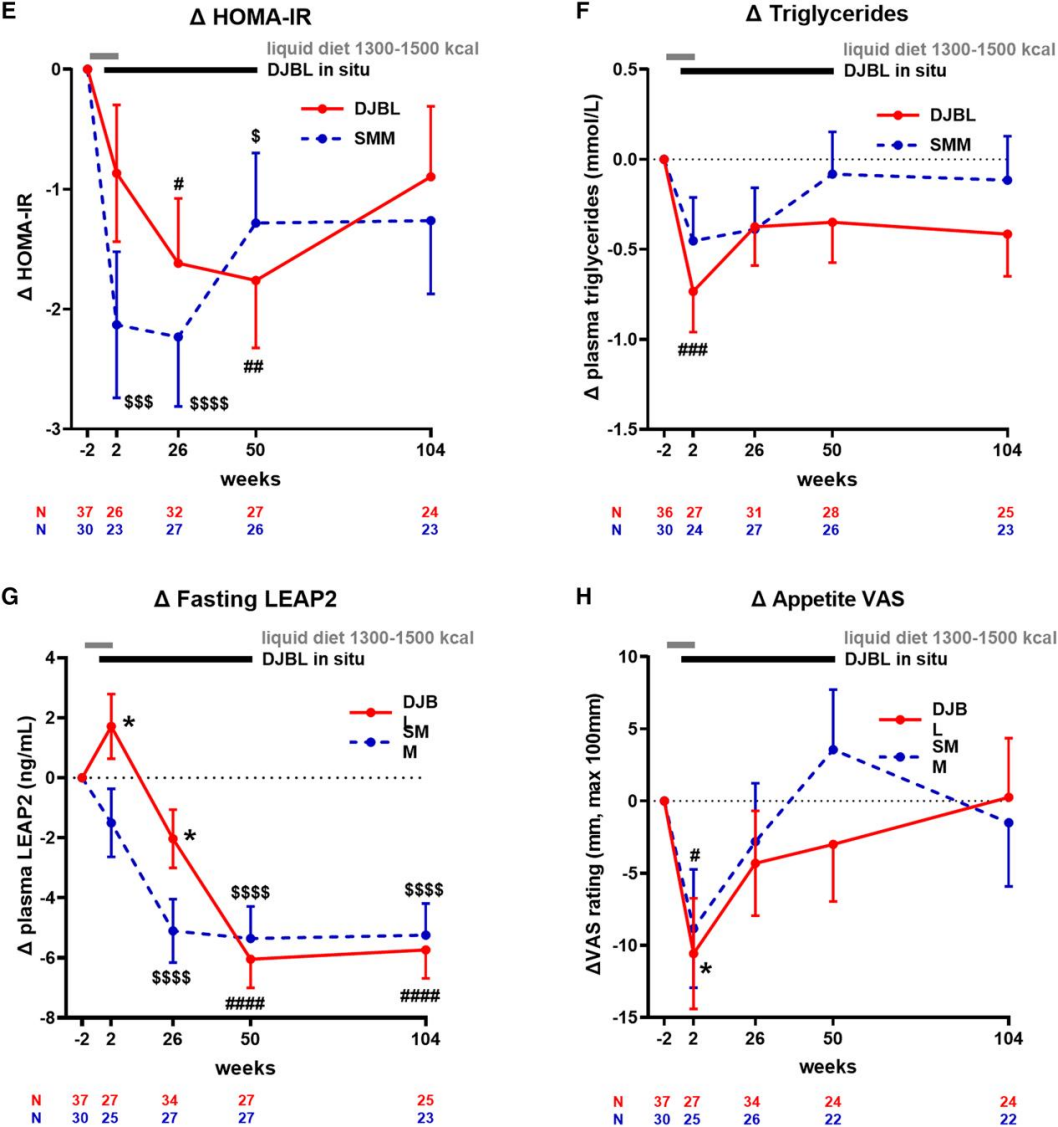
Longitudinal analysis of body mass index (BMI) and change in percentage weight, glycated hemoglobin A_1c_ (HbA_1c_), fasting glucose, HOMA-IR, fasting triglycerides, fasting plasma liver/foregut satiety hormone liver-expressed antimicrobial peptide 2 (LEAP2), and appetite ratings across visits. A, BMI, change in B, percentage weight loss; C, HbA_1c_; D, fasting glucose; E, HOMA-IR; F, fasting triglycerides; G, fasting plasma LEAP2; and H, fasting appetite VAS ratings vs −2 weeks, across time for participants undergoing standard medical management alone (SMM, dashed line) and duodenal-jejunal bypass liner (DJBL, solid line) groups. Black bar indicates duration of DJBL being in situ (0-52 weeks). N indicates number of participants at each time point. Gray bar indicates duration of liquid diet (−1 to 2 weeks). Statistical results below graph indicates linear mixed model analysis including group (SMM, DJBL), sweet taste detection task (done in the taste subgroup at weeks −2, 2, and 26 only, for fasting glucose, HOMA-IR, and fasting LEAP2 only) as between-participant, and weeks (−2, 2, 26, 50, and 104) as within-participant fixed factors, to examine effects of interventions on dependent variables over time. DJBL vs SMM: *P less than .05, **P less than .01, ***P less than .005, ****P less than .001; DJBL vs baseline week −2: # *P* less than .05, ## *P* less than .01, ### *P* less than .005, #### *P* less than .001; SMM vs baseline week −2: $ *P* less than .05, $$ *P* less than .01, $$$ *P* less than .005, $$$$  *P* less than .001. All data given as mean ± SEM. Abbreviations: HOMA-IR, homeostatic model assessment for insulin resistance; kcal, kilocalorie; VAS, visual analogue scale.

Including weeks (−2, 2, 26, 50, 104) as a within-participant factor, there was a statistically significant effect of weeks on absolute BMI (*P* < .001) and change in percentage weight (*P* < .001) (Fig. 1A and 1B; Supplementary Table S5 [63]). In post hoc analysis, absolute BMI decreased and remained lower than baseline at 104 weeks (effect size −2.04 ± 0.41 kg/m^2^; *P* < .001). The greatest loss in percentage weight occurred at 26 weeks (effect size −8.09 ± 0.99%; *P* < .001), and remained below baseline at 104 weeks (effect size −6.35 ± 1.04%; *P* < .001) (see Fig. 1A and 1B, Supplementary Table S6 [63]).

##### Duodenal-jejunal bypass liner group

Including weeks (−2, 2, 26, 50) as a within-participant factor, there was a statistically significant effect of weeks on absolute BMI (*P* < .001) and change in percentage weight (*P* < .001) (see Fig. 1A and 1B; Supplementary Table S7 [63]), with absolute BMI decreasing and remaining lower than baseline at 50 weeks (effect size −4.23 ± 0.30; *P* < .001). The greatest percentage weight loss occurred at 50 weeks (effect size −11.35 ± 0.70%; *P* < .001), with subsequent weight regain after DJBL explant (see Fig. 1A and 1B, Supplementary Table S8 [63]).

##### Duodenal-jejunal bypass liner vs standard medical management group

Including weeks (−2, 2, 26, 50, 104) as a within-participant factor and group as a between-participant factor, there was a statistically significant group × weeks interaction for BMI (*P* = .004) and percentage weight change (*P* = .002) (see Fig. 1A and 1B; Supplementary Tables S9, S11 [63]). DJBL participants lost significantly more weight than SMM participants at only weeks 26 and 50 (DJBL vs SMM; 26 weeks: effect size −2.20 ± 1.04%; *P* = .036; 50 weeks: effect size −3.44 ± 1.09%; *P* = .002) (see Fig. 1A and 1B, Supplementary Tables S10, S12 [63]).

#### Longitudinal changes in fasting glucose metabolism, triglycerides, and appetite

There was no statistically significant group × weeks interaction nor effect of group, but a significant effect of weeks on changes in HbA_1c_, fasting plasma glucose, HOMA-IR, serum triglycerides, and appetite VAS ratings (Fig. 1C-1F and 1H, Supplementary Tables S11 and S12 [63]); see Supplementary Results 3.2.2 for more details [63].

#### Longitudinal changes in fasting plasma LEAP2

##### Standard medical management group

There was a statistically significant effect of weeks on change in plasma LEAP2 (*P* < .001) (Supplementary Fig. S2, Supplementary Table S5 [63]), with the largest change in plasma LEAP2 at week 26 (effect size −3.78 ± 0.81 ng/mL; *P* < .001), remaining below baseline at week 104 (effect size −1.83 ± 0.86 ng/mL; *P* = .019) (see Fig. 1G and Supplementary Fig. S2, Supplementary Table S6 [63]).

##### Duodenal-jejunal bypass liner group

There was a statistically significant effect of weeks on changes in plasma LEAP2 (*P* < .001) (Supplementary Fig. S2 and Supplementary Table S7 [63]), with the largest increase in plasma LEAP2 at week 2 (effect size +1.98 ± 0.96 ng/mL; *P* = .042). The largest decrease in plasma LEAP2 occurred at week 50 (effect size −2.44 ± 1.00 ng/mL; *P* = .017) (see Fig. 1G and Supplementary Fig. S2, Supplementary Table S8 [63]).

##### Duodenal-jejunal bypass liner vs standard medical management group

There was a statistically significant group × weeks interaction for absolute plasma LEAP2 (*P* = .003) (Supplementary Fig. S2, Supplementary Table S9 [63]), with absolute plasma LEAP2 significantly higher in DJBL vs SMM at week 2 (effect size +2.88 ± 1.23 ng/mL; *P* = .021) (see Fig. 1G and Supplementary Fig. S2, Supplementary Table S10 [63]).

There was a statistically significant group × weeks interaction for change in plasma LEAP2 (*P* = .039) (see Fig. 1G and Supplementary Table S11 [63]). Change in plasma LEAP2 was significantly higher in DJBL vs SMM at 2 weeks (effect size +3.22 ± 1.56 ng/mL; *P* = .041) and 26 weeks (effect size +3.07 ± 1.1.44 ng/mL; *P* = .034) (see Fig. 1G and Supplementary Table S12 [63]).

### Correlations of Fasting Plasma LEAP2 With Fasting Outcomes

#### Correlations with weight

##### Standard medical management group

**Figure 2.**
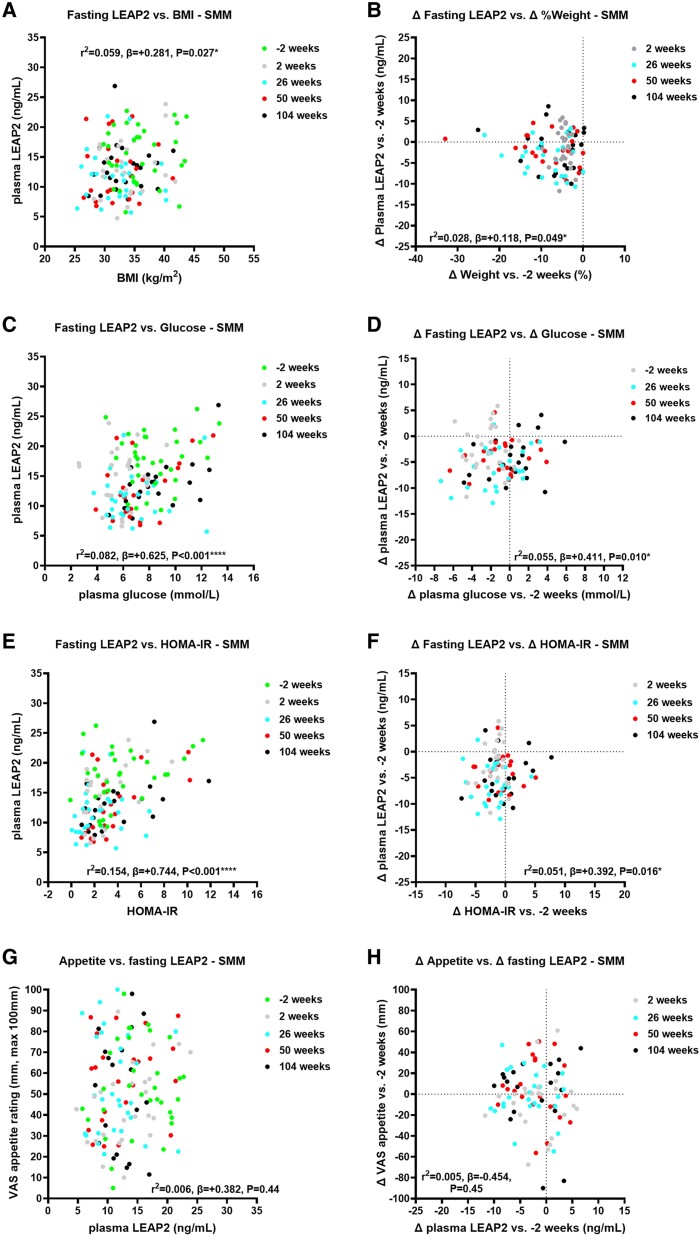
Correlations of absolute and change in fasting plasma liver/foregut satiety hormone liver-expressed antimicrobial peptide 2 (LEAP2) with absolute and change in weight, fasting glucose, HOMA-IR, and appetite ratings across visits in the SMM group. Correlations of absolute fasting plasma LEAP2 with A, body mass index (BMI); C, absolute fasting plasma glucose; E, HOMA-IR; G, absolute fasting appetite VAS ratings across visits −2, 2, 26, 50, and 104 weeks; and correlations of change in fasting plasma LEAP2 with B, percentage weight change; change in D, fasting plasma glucose; F, HOMA-IR; and H, fasting appetite VAS ratings between weeks 2, 26, 50, and 104 vs −2 weeks using linear mixed model analysis including sweet taste detection task (done in the taste subgroup at weeks −2, 2, and 26 only, for fasting glucose, HOMA-IR and LEAP2 only) as between-participant, and weeks (−2, 2, 26, 50, 104) as within-participant fixed factors, to examine effects of interventions on absolute and change in fasting plasma LEAP2 over time with pseudo *r*^2^ and β parameters. *P less than .05, **P less than .01, ***P less than .005, ****P less than .001. n = 22-30. Abbreviations: HOMA-IR, homeostatic model assessment for insulin resistance; SMM, standard medical management; VAS, visual analogue scale.

Over 104 weeks, absolute plasma LEAP2 positively correlated with BMI (Fig. 2A, Supplementary Table S13 [63]), and changes in plasma LEAP2 vs baseline also positively correlated with change in percentage weight (Fig. 2B, Supplementary Table S13 [63]).

##### Duodenal-jejunal bypass liner group

**Figure 3.**
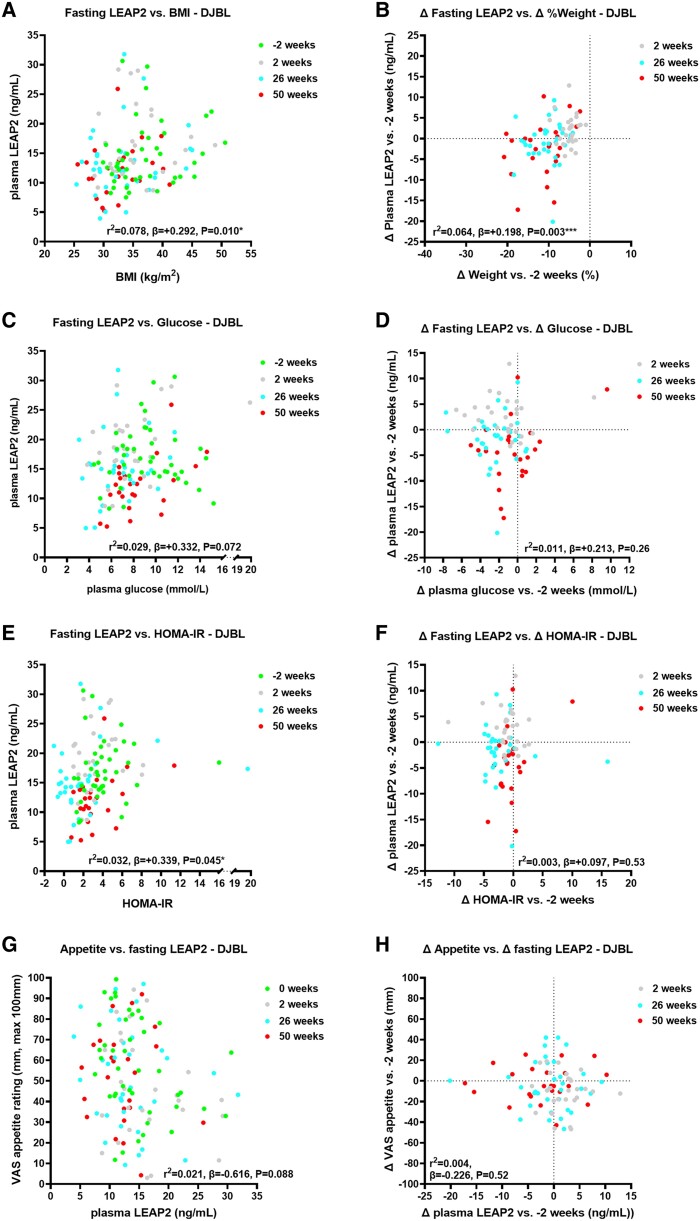
Correlations of absolute and change in fasting plasma liver/foregut satiety hormone liver-expressed antimicrobial peptide 2 (LEAP2) with absolute and change in weight, fasting glucose, HOMA-IR, and appetite ratings across visits in the DJBL group. Correlations of absolute fasting plasma LEAP2 with A, body mass index (BMI); C, absolute fasting plasma glucose; E, HOMA-IR; and G, absolute fasting appetite VAS ratings across visits −2, 2, 26, and 50 weeks; and correlations of change in fasting plasma LEAP2 with B, percentage weight change; change in D, fasting plasma glucose; F, HOMA-IR; and H, fasting appetite VAS ratings between weeks 2, 26, and 50 vs −2 weeks using linear mixed model analysis including sweet taste detection task (done in the taste subgroup at weeks −2, 2, and 26 only, for fasting glucose, HOMA-IR and LEAP2 only) as between-participant, and weeks (−2, 2, 26, 50) as within-participant fixed factors, to examine effects of interventions on absolute and change in fasting plasma LEAP2 over time with pseudo *r*^2^ and β parameters. *P less than .05, **P less than .01, ***P less than .005, ****P less than .001. n = 22-30. Abbreviations: DJBL, duodenal-jejunal bypass liner; HOMA-IR, homeostatic model assessment for insulin resistance; VAS, visual analogue scale.

Over 50 weeks, absolute plasma LEAP2 positively correlated with BMI (Fig. 3A, Supplementary Table S15 [63]), and changes in plasma LEAP2 vs baseline also positively correlated with percentage weight change (Fig. 3B, Supplementary Table S15 [63]).

##### Duodenal-jejunal bypass liner vs standard medical management group

In comparison of correlational analysis between groups (ie, difference in β parameters) over 50 weeks, there was no statistically significant difference in correlations of absolute or changes in plasma LEAP2 with BMI between groups (*P* = .38-.97) (Supplementary Tables S18 and S19 [63]).

#### Correlations with fasting glucose metabolism

##### Standard medical management group

Absolute plasma LEAP2 positively correlated with plasma glucose, HOMA-IR (Fig. 2C and 2E, Supplementary Fig. S5A and Supplementary Tables S13, S17 [63]), HbA_1c_, and serum insulin (Supplementary Fig. S3A and S3C, Supplementary Table S14 [63]). Changes in plasma LEAP2 also positively correlated with changes in plasma glucose, HOMA-IR (Fig. 2D and 2F, Supplementary Tables S13 and S17 [63]), and HbA_1c_, but not with changes in serum insulin (Supplementary Fig. S3B and S3D, Supplementary Table S14 [63]).

##### Duodenal-jejunal bypass liner group

Absolute plasma LEAP2 positively correlated with HOMA-IR and HbA_1c_ (Fig. 3E, Supplementary Figs. S4A and S5B, and Supplementary Tables S15-S17 [63]). Absolute plasma LEAP2 tended to positively correlate with absolute plasma glucose and serum insulin (Fig. 3C and Supplementary Fig. S4C and Supplementary Tables S15 and S16 [63]). Changes in plasma LEAP2 did not correlate with changes in plasma glucose, HOMA-IR, nor serum insulin (Fig. 3D and 3F and Supplementary Fig. S4D and Supplementary Tables S15 and S16 [63]), but positively correlated with changes in HbA_1c_ (Supplementary Fig. S4B and Supplementary Table S16 [63]).

##### Duodenal-jejunal bypass liner vs standard medical management group

Over 50 weeks, there was a statistically significant between-group difference in correlation of absolute plasma LEAP2 with absolute HOMA-IR, with the β parameter being higher in the SMM than in the DJBL group (Supplementary Fig. S5A and S5B and Supplementary Table S18 [63]), but not for change in plasma LEAP2 and HOMA-IR (Supplementary Table S19 [63]). There were no between-group differences in correlations of absolute or changes in plasma LEAP2 with plasma glucose, serum insulin, nor HbA_1c_ (see Supplementary Tables S18 and S19 [63]).

#### Correlations with fasting triglycerides

##### Standard medical management group

Absolute plasma LEAP2 positively correlated with serum triglycerides (Supplementary Fig. S3E and S5C and Supplementary Tables S14 and S17 [63]). Change in plasma LEAP2 did not correlate with change in serum triglycerides (Supplementary Fig. S3F and Supplementary Table S14 [63]).

##### Duodenal-jejunal bypass liner group

Absolute or changes in plasma LEAP2 did not correlate with absolute or changes in serum triglycerides (Supplementary Figs. S4E and S4F, S5D, and Supplementary Tables S16 and S17 [63]).

##### Duodenal-jejunal bypass liner vs standard medical management group

Over 50 weeks, there was a statistically significant between-group difference in absolute plasma LEAP2 correlation with absolute serum triglycerides (see Supplementary Table S18 [63]), but not in changes in plasma LEAP2 with changes in serum triglycerides (see Supplementary Table S19 [63]).

#### Correlations with fasting appetite and hunger ratings

In the SMM group, there was no correlation between absolute or changes in appetite with absolute or changes in plasma LEAP2, even when including absolute or changes in glucose as a covariate (Figs. 2G and 2H, Supplementary Table S13 [63]).

In the DJBL group, there was a statistically significant negative correlation of absolute appetite with absolute LEAP2 when adjusting for plasma glucose (*r*^2^ = 0.074; *P* = .048; differences in slopes DJBL vs SMM; *P* = .063 (Fig. 3G; Supplementary Tables S15 and S18 [63]). There was a significantly more negative correlation of absolute appetite with absolute plasma LEAP2 in DJBL vs SMM groups (*P* = .042; see Supplementary Table S18 [63]). There was no statistically significant correlation between changes in appetite with changes in LEAP2, with or without changes in glucose as a covariate (*P* = .85-.93; Fig. 3H; see Supplementary Table S15 [63]), and no statistically significant group difference (see Supplementary Table S19 [63]).

There were similar findings for correlations between absolute or changes in hunger with absolute or changes in fasting LEAP2 (see Supplementary Tables S14, S16, S18, and S19 [63]); see Supplementary Results 3.3.4 for further details [63].

#### Correlation analysis of fasting plasma LEAP2 with outcome variables after duodenal-jejunal bypass liner explant

There were no statistically significant correlations between changes in plasma LEAP2 with changes in weight, HbA_1c_, plasma glucose, serum insulin, HOMA-IR, serum triglycerides, appetite, and hunger, during weight regain after DJBL explant from 50 to 104 weeks (Supplementary Fig. S6, Supplementary Table S20 [63]); see Supplementary Results 3.3.5 for further details [63].

### Longitudinal Study of Postprandial Variables

#### Longitudinal analysis of weight in postprandial subgroup

**Figure 4.**
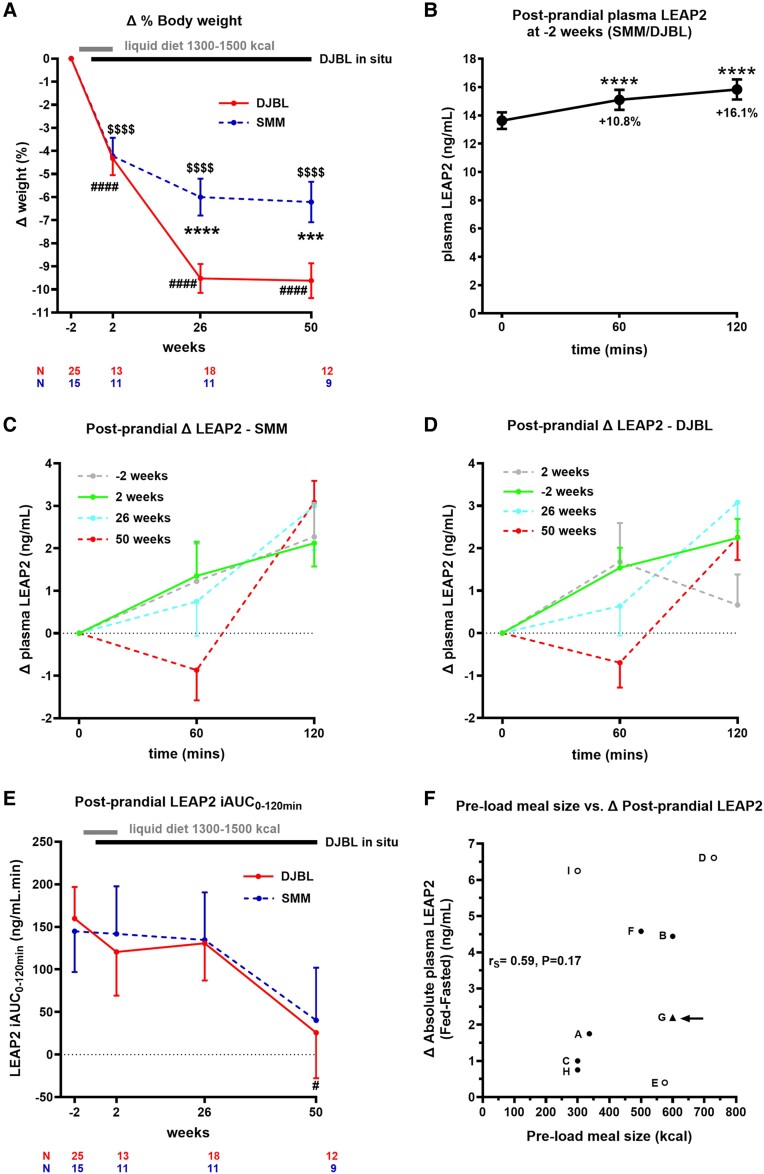
Changes in postprandial plasma liver/foregut satiety hormone liver-expressed antimicrobial peptide 2 (LEAP2). A and E, Longitudinal analysis of A, percentage weight loss and E, postprandial plasma LEAP2 iAUC from *t* = 0 minutes to *t* = 120 minutes after consumption of a fixed 600-kcal test meal (49% carbohydrate, 35% fat, 16% protein) across visits for participants undergoing standard medical management alone (SMM, dashed line) and duodenal-jejunal bypass liner (DJBL, solid line) groups. Black bar indicates duration of DJBL being in situ (0-52 weeks). Gray bar indicates duration of liquid diet (−1 to 2 weeks). N indicates number of participants at each time point. Statistical results below graph indicates linear mixed model analysis including group (SMM, DJBL), sweet taste detection task (done at weeks −2, 2, and 26 only), as between-participant, and weeks (−2, 2, 26, 50) as within-participant fixed factors, to examine effects of interventions on dependent variables over time: DJBL vs SMM *P less than .05, **P less than .01, ***P less than .005, ****P less than .001; DJBL vs baseline week 0: # *P* less than .05, ## *P* less than .01, ### *P* less than .005, #### *P* less than .001; SMM vs baseline week 0: $ *P* less than .05, $$ *P* less than .01, $$$ *P* less than .005, $$$$  *P* less than .001. B, Postprandial plasma LEAP2 after 600-kcal fixed test meal (49% carbohydrate, 35% fat, 16% protein) at baseline −2 week visit. Comparisons made using one-way repeated-measures analysis of variance including time (*t* = 0, 60, 120 minutes) as within-participant factors with post hoc Fisher least significant difference: *P less than .05, **P less than .01, ***P less than .005, ****P less than .001, *t* = 60 or 120 minutes vs 0 minutes, n = 40. C and D, Change in absolute postprandial plasma LEAP2 response at time points *t* = 60 and 120 minutes vs t = 0 minutes in C, DJBL group and D, SMM group, after 600-kcal fixed test meal at *t* = 0 minutes across visits. Statistical results given in Supplementary Tables S26-27 [63] made using linear mixed model analysis including time (*t* = 0, 60, 120 minutes) and weeks (−2, 2, 26, 50) as within-participant fixed factors, to examine effects of interventions on postprandial plasma LEAP2 over time. n = 9-25. F, Correlation of change in absolute plasma LEAP2 (ng/mL) concentrations between fed (+60 to +150 minutes) and fasted (0 minutes) time points (Δ fed-fasted) and preload meal size (kcal) consumed across different studies using Spearman (*r*_S_) correlation coefficient. Black dots indicate participants with obesity (BMI ≥30 kg/m^2^), black triangle with arrow indicates obesity with type 2 diabetes mellitus in the present study, open dots indicate participants without obesity (BMI < 30 kg/m^2^). Meals are nutritional supplements with similar macronutrient composition: carbohydrate: 49% to 55%, fat 30% to 35%, protein 14% to 18% energy. List of individual studies given in Supplementary Table S25 [63]. All data given as mean ± SEM. Abbreviations: BMI, body mass index; iAUC, incremental area under the curve; kcal, kilocalorie; OGTT, oral glucose tolerance test.

Over 50 weeks, there was a statistically significant group × weeks interaction, with the DJBL group losing significantly more weight than the SMM group at weeks 26 and 50 (Fig. 4A, Supplementary Tables S21 and S22 [63]); see Supplementary Results 3.4.1 for further details [63].

#### Postprandial response of plasma LEAP2

At −2 weeks, postprandial plasma LEAP2 response after a fixed test meal increased up to 2 hours after eating (see Fig. 4B; Supplementary Tables S23 and S24 [63]). In post hoc analysis, after food intake, plasma LEAP2 was 10.8% higher at time point *t* = 60 minutes (mean ± SEM, 15.10 ± 0.71 ng/mL) than at *t* = 0 minutes (13.63 ± 0.59 ng/mL) (effect size 1.47 ± 0.42 ng/mL [95% CI, 0.63-2.31]; *P* < .001), and 16.1% higher at *t* = 120 minutes (15.83 ± 0.72 ng/mL) (effect size 2.20 ± 0.34 ng/mL [95% CI, 1.50-2.89]; *P* < .001) (see Fig. 4B; Supplementary Table S24 [63]).

Comparing results across different human studies (overall 3 without obesity, 6 with obesity, including the present study) [17, 34, 36, 43, 44, 50, 51], there was a suggestion that the absolute postprandial increase in plasma LEAP2 is proportional to the preload fixed meal energy intake (*r*_S_ = +0.59; *P* = .17) (see Fig. 4F, Supplementary Table S25 [63]).

#### Longitudinal analysis of postprandial plasma LEAP2 response

##### Standard medical management group

Over 50 weeks, there was no statistically significant postprandial time point × visit interaction on postprandial plasma LEAP2 (Supplementary Table S26 [63]). There was a statistically significant main effect of time point (*P* < .001), but no significant main effect of visit (*P* = .81) on postprandial plasma LEAP2 (see Supplementary Table S26 [63]). In post hoc analysis, there was a trend for the change in postprandial plasma LEAP2 response at *t* = 120 minutes to be greater at 50 weeks vs baseline (effect size +0.96 ± 0.50 ng/mL; *P* = .092) (see Fig. 4C and Supplementary Table S27 [63]).

##### Duodenal-jejunal bypass liner group

Over 50 weeks, there was a statistically significant time point × visit interaction (*P* = .036) on postprandial plasma LEAP2 (see Supplementary Table S26). In post hoc analysis, the change in postprandial plasma LEAP2 response was significantly lower at *t* = 60 minutes at 50 weeks vs baseline (effect size −2.24 ± 0.85 ng/mL; *P* = .024) (see Fig. 4D, Supplementary Table S27 [63]).

#### Longitudinal analysis of postprandial LEAP2 incremental area under curve

##### Standard medical management group

Over 50 weeks, there was no statistically significant effect of visit on plasma LEAP2 iAUC_0–120min_ (Supplementary Table S21 [63]). In post hoc analysis, there was no statistically significant difference in plasma LEAP2 iAUC_0–120min_ between visits (see Fig. 4E and Supplementary Table S22 [63]). Across all visits, there was no statistically significant correlation between the plasma LEAP2 iAUC_0–120min_ and BMI (mean ± SEM β = +7.72 ± 7.73 ng/mL.min; *P* = .32).

##### Duodenal-jejunal bypass liner group

Over 50 weeks, there was no statistically significant effect of visit on plasma LEAP2 iAUC_0–120min_ (Supplementary Table S21 [63]). In post hoc analysis, the largest decrease in plasma LEAP2 iAUC_0–120min_ was at week 50 (−134.05 ± 65.11 ng/mL.min; *P* = .042) (see Fig. 4E, Supplementary Table S22 [63]). Across all visits, there was no statistically significant correlation between the plasma LEAP2 iAUC_0–120min_ and BMI (mean ± SEM β = −4.06 ± 4.22 ng/mL.min; *P* = .34).

##### Duodenal-jejunal bypass liner vs standard medical management *group*

There was no statistically significant group × visit interaction nor significant main effect of visit or group on plasma LEAP2 iAUC_0–120min_ (Supplementary Table S21 [63]). In exploratory post hoc analysis, there was no group difference in plasma LEAP2 iAUC_0–120min_ at any visit, nor any statistically significant change in plasma LEAP2 iAUC_0–120min_ from baseline in either the SMM or DJBL group, other than a significant decrease in the DJBL group at 50 weeks when there was a 9.6% weight loss (see Fig. 4E and Supplementary Table S22 [63]). In the DJBL group, percentage weight loss from baseline to 50 weeks did not positively correlate with the decrease in plasma LEAP2 iAUC_0–120min_ from baseline to 50 weeks (*P* = .38).

#### Longitudinal analysis of postprandial appetite and fullness ratings

There was no statistically significant main effect of week in within-group or between-group analysis of appetite iAUC_0–120min_, but there was significant effect of week within the DJBL group and hence between group analysis of fullness iAUC_0–120min_ (Supplementary Fig. S7A and S7B; Supplementary Tables S28 and S29 [63]); see Supplementary Results 3.4.5 for further details [63].

#### Correlation with postprandial appetite and fullness ratings

In within-group and between-group analysis, there were no statistically significant correlations with absolute and change in appetite iAUC_0–120min_ or fullness iAUC_0–120min_ with absolute and change in plasma LEAP2 iAUC_0–120min_, even when including plasma glucose as a covariate (Supplementary Fig. S7C-S7F and Supplementary Table S30 [63]); see Supplementary Results 3.4.6 for further details [63].

## Discussion

In this longitudinal human study of adults with obesity and inadequately controlled T2DM, the following results were found (see Table 2): (i) fasting plasma LEAP2 was reduced over 2 years with weight loss and improved glycemic control via LCD, increased physical activity, and standard pharmacological management of T2DM (with rare use of GLP-1 analogues); (ii) fasting plasma LEAP2 over this 2-year period through lifestyle modification was positively correlated with absolute BMI, fasting plasma glucose, HbA_1c_, serum insulin and triglycerides, and HOMA-IR for absolute plasma LEAP2, and positively correlated with decreases in weight, fasting plasma glucose, HbA_1c_, and HOMA-IR for decreases in plasma LEAP2; (iii) after DJBL insertion fasting plasma LEAP2 had a delayed and smaller decrease up to 26 weeks, and at 50 weeks was similar to the control group, despite the greater weight loss and similar decreases in fasting plasma glucose, HbA_1c_, serum insulin, HOMA-IR, and serum triglycerides; (v) fasting plasma LEAP2 over this 1-year period through DJBL insertion in addition to lifestyle modification was positively correlated with absolute BMI, HbA_1c_, and HOMA-IR for absolute plasma LEAP2, and positively correlated with decreases in weight and HbA_1c_ for decreases in plasma LEAP2; (vi) there was no statistically significant differences in the relationships between absolute or decreases in fasting plasma LEAP2 with BMI, weight, fasting plasma glucose, or HbA_1c_ or serum insulin, though there were more positive correlations of absolute plasma LEAP2 with absolute HOMA-IR and fasting serum triglycerides (though not for decreases in variables) for the DJBL group than the SMM alone group; (vii) weight regain 1 year after explant of DJBL was not associated with any statistically significant increase in fasting plasma LEAP2; (viii) although postprandial plasma LEAP2 increased after a fixed 600-kcal liquid meal at baseline, with a 16.1% increase at 2 hours, there were no changes in the postprandial increase in plasma LEAP2 with weight loss and improved glycemic control over time in either group, nor any statistically significant difference in the postprandial increase in plasma LEAP2 between groups; and (ix) there were no statistically significant correlations between absolute or changes in fasting or postprandial plasma LEAP2 and absolute or changes in hunger, fullness, or appetite in either group, nor differences in these relationships between the groups.

**Table 2.** Summary table of correlational results

Summary of results						
Outcome variable		LEAP2	SMM	DJBL	DJBL vs SMM	Post-DJBL explant
**Fasting**	**BMI**	**Absolute**	**+**	**+**	o	n/a
	**% Weight**	**Delta (Δ)**	**+*^a^***	**+*^b^***	o	o*^c^*
	**Plasma glucose**	**Absolute**	**+**	o	o	n/a
		**Delta (Δ)**	**+*^a^***	o*^b^*	o*^b^*	o*^c^*
	**Serum insulin**	**Absolute**	**+**	o	o	n/a
		**Delta (Δ)**	o*^a^*	o*^b^*	o*^b^*	o*^c^*
	**Serum triglycerides**	**Absolute**	**+**	o	**↑**	n/a
		**Delta (Δ)**	o*^a^*	o*^b^*	o*^b^*	o*^c^*
	**HbA_1c_**	**Absolute**	**+**	**+**	o	n/a
		**Delta (Δ)**	**+*^a^***	**+*^b^***	o*^b^*	o*^c^*
	**HOMA-IR**	**Absolute**	**+**	**+**	**↑**	n/a
		**Delta (Δ)**	**+*^a^***	o*^b^*	o*^b^*	o*^c^*
	**Appetite VAS ratings**	**Absolute**	o	o	o	n/a
		**Delta (Δ)**	o*^a^*	o*^b^*	o*^b^*	o*^c^*
	**Hunger VAS ratings**	**Absolute**	o	o	o	n/a
		**Delta (Δ)**	o*^a^*	o*^b^*	o*^b^*	o*^c^*
**Postprandial**	**Appetite VAS ratings**	**Absolute*^d^***	o	o	o	n/a
		**Delta (Δ)*^d^***	o*^b^*	o*^b^*	o*^b^*	n/a
	**Fullness VAS ratings**	**Absolute*^d^***	o	o	o	n/a
		**Delta (Δ)*^d^***	o*^b^*	o*^b^*	o*^b^*	n/a

This table shows the correlation of absolute and changes in fasting or postprandial plasma LEAP2 with absolute and changes in BMI, plasma glucose, serum insulin, triglycerides, HbA_1c_, HOMA-IR, appetite, hunger, and fullness VAS ratings for SMM and DJBL groups and the differences between groups (DJBL vs SMM) across visits. All changes expressed as delta (Δ). + indicates significant positive correlation, o indicates no correlation, ↑ indicates significantly greater correlation in DJBL vs SMM groups.

Abbreviations: BMI, body mass index; DJBL, duodenal-jejunal bypass liner; HbA_1c_, glycated hemoglobin A_1c_; HOMA-IR, Homeostatic Model Assessment for Insulin Resistance; iAUC, incremental area under the curve; n/a, not applicable; SMM, standard medical management; VAS, visual analogue scale.

^*a*^Δ 104 vs −2 weeks.

^*b*^Δ 50 vs −2 weeks.

^*c*^Δ 104 vs 50 weeks.

^*d*^Postprandial variables expressed as iAUC from *t* = 0 minutes to *t* = 120 minutes.

### Decreases in Plasma LEAP2 During Weight Loss

To our knowledge, this is the longest study finding a decrease in fasting plasma LEAP2 with LCD (500 kcal per day deficit) with increased physical activity–induced weight loss in obesity (with T2DM) over 2 years in the SMM group. These findings are consistent with the previous literature of shorter-term caloric restriction and weight loss. Acute overnight fasting lowers fasting plasma LEAP2 in mice and humans [17] and 36-hour fasting in humans [50]. In mice with normal weight, (i) a 40% caloric restriction over 2 weeks tended to decrease fasting plasma LEAP2 [31], while (ii) weight loss in diet-induced obesity (high-fat diet for 12 weeks) after return to normal chow for 4 weeks [17], (iii) a 50% caloric restriction over 14 days in mice eating normal chow [64], and (iv) a ketogenic diet with 75% dietary fat over 3 weeks leading to an approximately 14% weight loss in chow-fed mice [44], all decreased fasting plasma LEAP2. In humans, short-term 2-week LCD (1200 kcal/day) without and with interval exercise (60 minutes/day) in women with obesity lowered fasting plasma LEAP2, and this was likely due to caloric restriction and weight loss, since exercise had no additive effect [51].

Weight loss from bariatric surgery also lowers fasting plasma LEAP2. In mice, duodenal *Leap2* messenger RNA (mRNA) expression decreased after VSG surgery [31]. In humans, weight loss after VSG and RYGB surgery for obesity reduced fasting and/or postprandial plasma LEAP2 at 3 to 24 months after 16% to 31% weight loss [17, 34].

In the present study, postprandial plasma LEAP2 increased after a 600-kcal liquid meal at baseline in adults with obesity and T2DM, consistent with the previous literature [17, 42, 43, 50]. In mice, the magnitude of postprandial increases in plasma LEAP2 increased with greater meal size [45]. Adding the present study to our previous analysis across different human studies [42], there is a suggestion that postprandial increases in plasma LEAP2 are proportional to the caloric value of the preload meal (see Fig. 4F), despite slight differences in macronutrient content and the timing of the postprandial sampling.

Surprisingly, in the present study, there was no decrease in postprandial increases in plasma LEAP2 with weight loss and improved glycemic/metabolic control from LCD and increased physical activity–induced weight loss in obesity and T2DM over 2 years in the SMM group; however, this was examined only in a smaller subset of the cohort. Similarly, there was only a trend for the postprandial increase in plasma LEAP2 to be attenuated at 12 to 18 months after VSG surgery, and no statistically significant change in the postprandial increase in plasma LEAP2 at 3 months after RYGB surgery [17].

This suggests that decreases in plasma LEAP2 may be driven by weight loss or reduced food intake, rather than a secondary consequence of the specific and different anatomical gut manipulations from RYGB and VSG surgery. However, it cannot be excluded that the decreases in fasting plasma LEAP2 are due to improvement in glycemic control, metabolic syndrome, and insulin resistance. Fasting plasma/serum LEAP2 did not fall significantly after RYGB surgery in obesity with metabolic dysfunction–associated fatty liver disease (MAFLD) at 6 months with an approximately 29% weight loss despite marked improvements in insulin resistance and fasting plasma glucose [65], nor after VSG surgery in obesity with T2DM at 12 months with an approximately 28% weight loss despite marked improvements in glycemic control and markers of metabolic syndrome [66]. However, after VSG surgery for T2DM, the decreases in BMI, fasting serum triglycerides, but not HbA_1c_, did correlate with decreases in serum LEAP2 [66].

In the present study, in the SMM group with obesity and T2DM, BMI positively correlated with fasting plasma LEAP2 across all visits, both for absolute values and decreases from baseline visit. Several single time point cross-sectional studies have also found positive correlations between fasting plasma LEAP2 and BMI, in adults across a range of BMIs from normal weight to obesity, mainly without T2DM [17], overweight/obesity and T2DM [43, 66], overweight with MAFLD [46], obesity and prediabetes [67], and with percentage body fat, visceral adiposity, and liver fat [17, 46, 67], though these positive correlations with BMI or percentage body fat were not seen in one study of adults with overweight/obesity [48]. No previous studies of weight loss after bariatric surgery or LCD have reported correlations of changes in BMI or weight with changes in fasting plasma LEAP2 [17, 51].

In the present study in obesity and T2DM, there was no correlation between BMI and the postprandial increase in plasma LEAP2 across all visits over 50 weeks in the subgroup having the fixed meal, in contrast to other studies reporting that higher BMI is associated with lower postprandial increases in plasma LEAP2 in adults without obesity or T2DM [42], but with higher postprandial plasma LEAP2 across 2 discrete groups with normal weight and obesity without T2DM [17].

### Fasting Plasma LEAP2 and Glucose and Fat Metabolism

In the present study of obesity with T2DM, fasting plasma LEAP2 over the 2-year period in the SMM group was positively correlated with fasting plasma glucose, HbA_1c_, serum insulin and triglycerides, and HOMA-IR for absolute plasma LEAP2, and decreases in plasma LEAP2 positively correlated with decreases in fasting plasma glucose, HbA_1c_, and HOMA-IR.

In support of these findings, both in mice without obesity and in humans with obesity, fasting/postprandial plasma LEAP2 positively correlated with fasting/postprandial plasma glucose [17], while fasting plasma LEAP2 positively correlated with fasting plasma glucose, serum insulin, and/or HOMA-IR, in adults with normal weight/overweight/obesity [17], with obesity [43, 48], in overweight/obesity with prediabetes [67], in adults with MAFLD without obesity [46], though not in adults without obesity, without T2DM [42] or with T2DM [47]. Furthermore, fasting plasma LEAP2 positively correlated with fasting serum triglycerides in adults with normal weight/overweight/obesity [17], and in overweight/obesity with prediabetes [67], but not in adults with T2DM without obesity [47], while postprandial increases in plasma LEAP2 tended to negatively correlate with postprandial increases in triglycerides in adults without obesity or T2DM [42].

This suggests that metabolic factors including blood glucose, insulin, and triglycerides may be mediators of plasma LEAP2 regulation. However, medications to lower plasma glucose and improve insulin resistance (including metformin and dapagliflozin) in adults with overweight/obesity and prediabetes did not decrease fasting plasma LEAP2 over 13 weeks [67].

Another potential mediator of weight loss–associated decreases in plasma LEAP2 is elevations of blood ketones as a result of lipolysis. In men without obesity, 60 minutes’ endurance exercise reaching 70% maximal oxygen consumption lowered fasting plasma LEAP2, associated with increases in plasma ketone β-hydroxybutyrate [44]. β-Hydroxybutyrate has been postulated to directly lower plasma LEAP2 through hepatic action, via studies examining effects on LEAP2 secretion from murine hepatocytes ex vivo, while ketogenic diets and exogenous ketone administration lowered plasma LEAP2 in animal studies and in men without obesity [44, 50], though the opposite effect on plasma LEAP2 was seen in another study of exogenous ketone administration in men without obesity [49]. Oral ingestion of the ketone β-hydroxybutyrate also suppresses plasma total ghrelin and AG [68, 69], while in the ketogenic phase before weight maintenance, VLCD suppresses or attenuates weight loss–associated increases in fasting plasma AG or total ghrelin [70-72]. Future work should examine correlations between plasma β-hydroxybutyrate and plasma LEAP2 in the present study, though the intervention was only an LCD not a VLCD.

### Plasma LEAP2 After Duodenal-Jejunal Bypass Liner Insertion

Endoscopic DJBL insertion mimics the exclusion of food from the duodenum and jejunum seen after RYGB surgery [55, 56, 59], though after RYGB there is also exclusion of food from the stomach remnant, and also surgical interference with vagal nerve signaling, and effects on bile salt flow, increases in satiety gut hormones PYY and GLP-1, and changes in gut microbiome [73, 74].

Weight loss after RYGB surgery for obesity without/with T2DM reduced fasting and/or postprandial plasma LEAP2 at 3 to 24 months after 16% to 31% weight loss [17, 34].

However, in the present study of obesity with T2DM, the decrease in fasting plasma LEAP2 with weight loss was delayed and attenuated after DJBL insertion compared to the SMM group. Indeed, at the first 2-week time point after DJBL insertion, fasting plasma LEAP2 had increased relative to baseline, while by 26 weeks fasting plasma LEAP2 had decreased relative to baseline, but the decrease was less than in the SMM group, while at 50 weeks the decrease in plasma LEAP2 was similar in the DJBL and SMM groups, despite weight loss being greater at 26 and 50 weeks in the DJBL than in the SMM groups, though improvements in HbA_1c_ were similar. This suggests a dissociation between weight loss and fasting plasma LEAP2 in the DJBL vs SMM groups, although there was no difference in the slopes of the relationship between absolute or change in fasting plasma LEAP2 and absolute BMI or percentage weight loss between the groups. However, there was a more positive correlation of fasting plasma LEAP2 with fasting serum triglycerides and HOMA-IR in the DJBL vs SMM groups, which could contribute to the attenuated decrease in fasting plasma LEAP2 in the DJBL group, despite the similar decreases in fasting serum triglycerides and HOMA-IR between the groups. However, the exact mechanisms behind this delayed and attenuated decrease in fasting plasma LEAP2 in the DJBL group remains unclear. It is important to appreciate that both groups had a 3-week 1300 to 1500 kcal per day diet (depending on sex) before the DJBL insertion or commencement of LCD.

One potential confounder is that the DJBL group, but not SMM group, received PPIs and *Helicobacter pylori* eradication therapy prior to the DJBL insertion, and the DJBL group had a greater prevalence of PPI usage at all visits from 2 to 50 weeks. This could theoretically influence plasma LEAP2, though any possible mechanism is unclear given that LEAP2 is released from the duodenum/ileum and liver rather than the stomach. There are no published data available on the effects of PPI use or *H pylori* presence or eradication on plasma LEAP2. However, there was no difference in fasting plasma LEAP2 at baseline (−2 weeks) across both groups between those not using PPIs (n = 33) or using PPIs (n = 10), without any covariates (*P* = .30), or including BMI or HOMA-IR as covariates (*P* = .63-.68). Of note, no differences have been seen in fasting serum total ghrelin in adults with vs without PPI use for 6 months [75], or in men including overweight/obesity in those with or without *H pylori* infection [76].

In the whole cohort, as with the 2 mechanistic subgroups presented in this paper, from the clinical trial of DJBL insertion vs SMM of obesity with inadequately controlled T2DM, there was greater weight loss in the DJBL group at 26 and 50 weeks, which was lost after DJBL explant at 104 weeks, with similar improvements in HbA_1c_ and fasting plasma glucose at 26, 50, and 104 weeks [56, 59]. The reason behind the greater weight loss in the DJBL group remains uncertain. There were no between-group differences in (i) the decrease in total food intake and macronutrient selection from 3-day food diaries and 1-day food recall, though there appeared to be a greater decrease in total energy intake and carbohydrate intake in the DJBL than in the SMM group from food frequency questionnaires (though this was higher at baseline in the DJBL than in the SMM group), nor changes in (ii) total food intake, intake of low/high fat, savory/sweet foods, or macronutrient intake at an ad libitum lunch meal when fasted overnight, (iii) sweet taste detection or hedonics, (iv) motivation for sweets from a progressive ratio task, (v) high-energy or low-energy food cue reactivity using functional magnetic resonance imaging, (vi) explicit liking/wanting and implicit wanting of low-/high-fat, savory/sweet foods using the Leeds Food Preference Questionnaire, (vii) pleasantness ratings of low-/high-fat, savory/sweet foods, (viii) eating behavior questionnaires assessing dietary restraint, hunger-related, external, disinhibited, and emotional eating, “food addiction” and binge-eating related behaviors, (ix) fasting and postprandial VAS ratings of hunger, fullness, pleasantness to eat, desired food intake, and nausea; and (x) fasting and postprandial satiety hormones PYY, GLP-1, and FGF-19, though sample sizes were low in some of these mechanistic subgroup outcomes [56, 58].

The delay in and attenuation of the decrease in fasting plasma LEAP2 with weight loss in the DJBL group could theoretically contribute to greater weight loss by attenuating the increase in GHSR signaling that results both from increased constitutive GHSR activity (with any decrease of LEAP2) and increase in plasma AG that occurs with weight loss from dietary restriction [11, 13-20]. However, this attenuation of GHSR signaling would be expected to result in blunting of weight loss–associated increases in appetite, food cue reactivity, or other eating behaviors, and greater reductions in energy intake, in the DJBL vs SMM groups, none of which were seen in this study, other than a possible greater reduction in total and carbohydrate intake from food frequency questionnaires, but not other measures [56].

One year after DJBL explant, there was no increase in fasting plasma LEAP2, despite rebound increases in weight from a nadir of 11.3% weight loss at 52 weeks to 5.1% at 104 weeks (though still remaining statistically significantly below baseline). Similar weight rebound was seen in the whole DJBL cohort after explant in the present study [56, 59], and in a previous study of adults with T2DM and obesity at 1 year after DJBL [77]. This suggests that there may be establishment of a new set point in fasting plasma LEAP2 after weight loss with DJBL insertion that may impair weight loss maintenance, since plasma LEAP2 did not increase with weight regain to assist with suppression of eating behavior. However, the effects of weight regain on plasma LEAP2 after dietary-induced weight loss alone needs to be examined, since in the SMM group weight loss was maintained between 52 and 104 weeks.

### Postprandial Increases in Plasma LEAP2

In the present study, postprandial LEAP2 increased after a 600-kcal liquid meal at baseline in adults with obesity and T2DM (across both groups), consistent with the previous literature [17, 42, 43, 50]. In preclinical studies, the magnitude of postprandial increases in plasma LEAP2 depends on the size and macronutrient content of meals, with greater increases with fats than carbohydrates [45]. Across human ingestion studies, despite differences in macronutrient content and timing of postprandial sampling, there is a suggestion that postprandial increases in LEAP2 are proportional to the caloric value of the preload meal with outliers including studies with adults without obesity (see [42]) and further expanded in the present study (Fig. 4F).

Contrary to the decrease in fasting plasma LEAP2 with weight loss in the SMM group, there was no statistically significant change in the postprandial increase in plasma LEAP2 after a fixed 600-kcal meal in the SMM group at 2, 26, or 50 weeks. Furthermore, despite the exclusion of food from the duodenum/jejunum, and their greater weight loss, there was also no change in the postprandial increase in plasma LEAP2 in the DJBL group at 2 nor 26 weeks, nor any difference from the SMM group. These negative findings may be complicated by the smaller sample sizes for those participants receiving the fixed meal. However, in exploratory analysis at 50 weeks in the DJBL group, the postprandial increase in plasma LEAP2 was attenuated compared to baseline when average weight loss across the 2 groups was at its maximum of 9.6%. This finding is consistent with the positive correlation of postprandial LEAP2 with BMI in a previous cross-sectional study in obesity [17], suggesting that postprandial increases in plasma LEAP2 are influenced by degree of obesity, being lower with lower BMI and after weight loss.

The lack of differences in the postprandial increases in plasma LEAP2 between the DJBL and SMM groups may suggest that the duodenum/jejunum is not vital in eliciting the postprandial increase in plasma LEAP2, with the liver being more important. In mice, both liver and jejunum/ileum *Leap2* mRNA expression decreased with fasting, but increased only in the liver after standard chow refeeding [39, 44], while mixed macronutrient ingestion (49% carbohydrate, 35% fat) increased both liver and jejunal *Leap2* mRNA expression [45]. Furthermore, in mice, olive, lard, and fish oil increased plasma LEAP2 though not liver *Leap2* mRNA, while only olive oil and lard increased jejunal *Leap2* mRNA, and olive oil but not fish oil increased LEAP2 secretion from jejunal organoids [45].

### Plasma LEAP2 and Appetite

Contrary to our hypothesis in the present study, there were no correlations between absolute/changes in fasting or postprandial plasma LEAP2 with absolute/changes in fasting hunger/appetite or postprandial fullness/appetite ratings in adults with obesity and T2DM in either the SMM or DJBL groups at baseline or across all visits, nor any differences between the groups. In previous studies, acute intravenous LEAP2 administration in men without obesity did not alter various appetite ratings, despite reducing ad libitum savory food intake [36]; in adults without obesity, postprandial increases in LEAP2 correlated positively with postprandial decreases in appetite but not increases in fullness ratings [42]; in adults with overweight/obesity, fasting plasma LEAP2 negatively correlated with fasting hunger ratings, but there was no correlation between absolute postprandial plasma LEAP2 and post-prandial hunger ratings [43]. The lack of any correlations in the present study may be contributed to by the longitudinal nature of the analysis and the fact that all participants had T2DM, though including plasma glucose as a covariate did not produce any significant findings.

### Strengths and Limitations

The novelty of the present study is the long duration of follow-up using 2 different mechanisms to achieve weight loss. Despite the expected participant dropouts over a longitudinal study, final sample sizes were reasonable although numbers for the postprandial plasm LEAP2 results were more limited. There was no sham endoscopic procedure in the SMM group, though the SMM group still received a stricter LCD intervention for 2 weeks as in the DJBL group around the time of implant, though the SMM group did not receive *H pylori* eradication therapy and long-term PPI medication.

A subset of the cohort had a sweet taste detection task prior to blood sampling, which had a significant cross-sectional effect to decrease fasting plasma LEAP2, but adjusted values to remove this effect were included in all analyses. Glycemic control was poor in all the T2DM participants at baseline due to the inclusion criteria of the trial, so results may be different in those with obesity without T2DM or with well-controlled T2DM.

Participants had their pharmacological treatment for T2DM changed during the trial following international guidelines, but there was no difference in use of medications between the groups. Furthermore, there were very few participants who started medications that might alter their appetite including GLP-1 receptor agonists and insulin.

### Future Directions

Given the increases in plasma total ghrelin and AG with dietary-induced weight loss [6, 11, 13-16, 18], which is also seen for total ghrelin in obesity and T2DM after DJBL insertion (though there was no dietary control group) [78], and the potential importance of the plasma AG/LEAP2 ratio in human eating behavior [17, 42], it will be important to measure plasma AG in this study. The effects of dietary-induced weight loss on fasting and plasma LEAP2 will also need to be assessed in people with obesity without T2DM and well-controlled T2DM.

### Conclusion

Dietary-induced weight loss with increased physical activity in participants with obesity and inadequately controlled T2DM caused fasting plasma LEAP2 decreases. Whether this may be due to weight loss and/or improved glycemic control and insulin sensitivity is uncertain, since absolute/decreases in fasting plasma LEAP2 positively correlated with absolute/decreases in BMI, HbA_1c_, fasting plasma glucose, serum insulin, HOMA-IR, and serum triglycerides. DJBL insertion in obesity/T2DM delayed and attenuated the weight loss–associated decrease in fasting plasma LEAP2 through uncertain mechanisms, which might have contributed to the greater weight loss after DJBL insertion, through attenuation of GHSR signaling, although differences in eating behavior or dietary intake between the 2 groups have not been identified. The lack of any differences in postprandial increases in plasma LEAP2 between the SMM and DJBL groups may implicate the liver rather than the duodenum/jejunum as the main source for postprandial secretion of LEAP2. The lack of any increase in fasting plasma LEAP2 following DJBL explant might represent the establishment of a novel set point after weight loss maintenance that may contribute to weight regain. These results add to our understanding of the role of plasma LEAP2 in human body weight regulation, though further longitudinal studies of weight loss are needed in adults with obesity without T2DM.

## Data Availability

Data generated during and/or analysed for this study are available from the corresponding author on reasonable request.

## Abbreviations

BMI: body mass index
DJBL: duodenal-jejunal bypass liner
GHSR: ghrelin receptor
GLP-1: glucagon-like peptide-1
HbA_1c_: glycated hemoglobin A_1c_
HOMA-IR: homeostatic model assessment for insulin resistance
iAUC: incremental area under the curve
LCD: low-calorie diet
LEAP2: liver-expressed antimicrobial peptide 2
LSD: least significant difference
MAFLD: metabolic dysfunction–associated fatty liver disease
mRNA: messenger RNA
PPI: proton pump inhibitor
RYGB: Roux-en-Y gastric bypass
SMM: standard medical management
T2DM: type 2 diabetes mellitus
VAS: visual analogue scale
VLCD: very low-calorie diet
VSG: vertical sleeve gastrectomy
